# Supplementary material for: RNA editing in cancer impacts mRNA abundance in immune response pathways
Source: Genome Biol. 2020 Oct 26;21:268. doi: 10.1186/s13059-020-02171-4 (PMC7586670; doi:10.1186/s13059-020-02171-4)
Supplement: Supplementary file 2 — Additional file 2: Fig. S1. Differential editing not confounded by metadata. Heatmaps of significance (log10-transformed adjusted p values) of correlations between the top two principal components and E/M phenotype among metadata fields in each cancer type. Darker color indicates smaller p value and stronger association. Fig. S2. Gene ontology enrichment among differentially edited genes. Significance of enrichment of gene ontology (GO) terms among all differentially edited genes (blue), only hyperedited genes (green) or only hypoedited genes (pink) of each cancer type. Point size represents the statistical significance of enrichment (log10-transformed adjusted p value). Terms significantly enriched in at least two cancer types are shown. For cancer types with a global hyperediting trend in M tumors, GO enrichment among hyperedited genes is similar to that among all differentially edited genes. Likewise, for cancer types with a hypoediting trend (BRCA and OV), enrichment among hypoedited genes is similar to that among all differentially edited genes. Fig. S3. Clustering of single cells from three lung cancer tumors. A. TSNE projection of cells based on expression profiles, with color indicating cluster identity (left). Cell types were assigned to clusters by matching differentially expressed genes of clusters to known cell type markers (right). B. TSNE projection of only cells from cluster 10 to further refine cell type assignment (left). Similar to A, cell types were labeled using differentially expressed genes that matched cell type markers (right). C. Counts of cells for each cell type after 2 rounds of clustering and cell type assignment (A and B). D. Log2-transformed expression values of marker genes across cell types. Signature matrix on the left indicates expression values assigned for each cell type by CIBERSORTx. On the right, Pooled Cells indicate that expression values were calculated from pooling reads from cells of the same type together. Fig. S4. E and [file 13059_2020_2171_MOESM2_ESM.pdf]

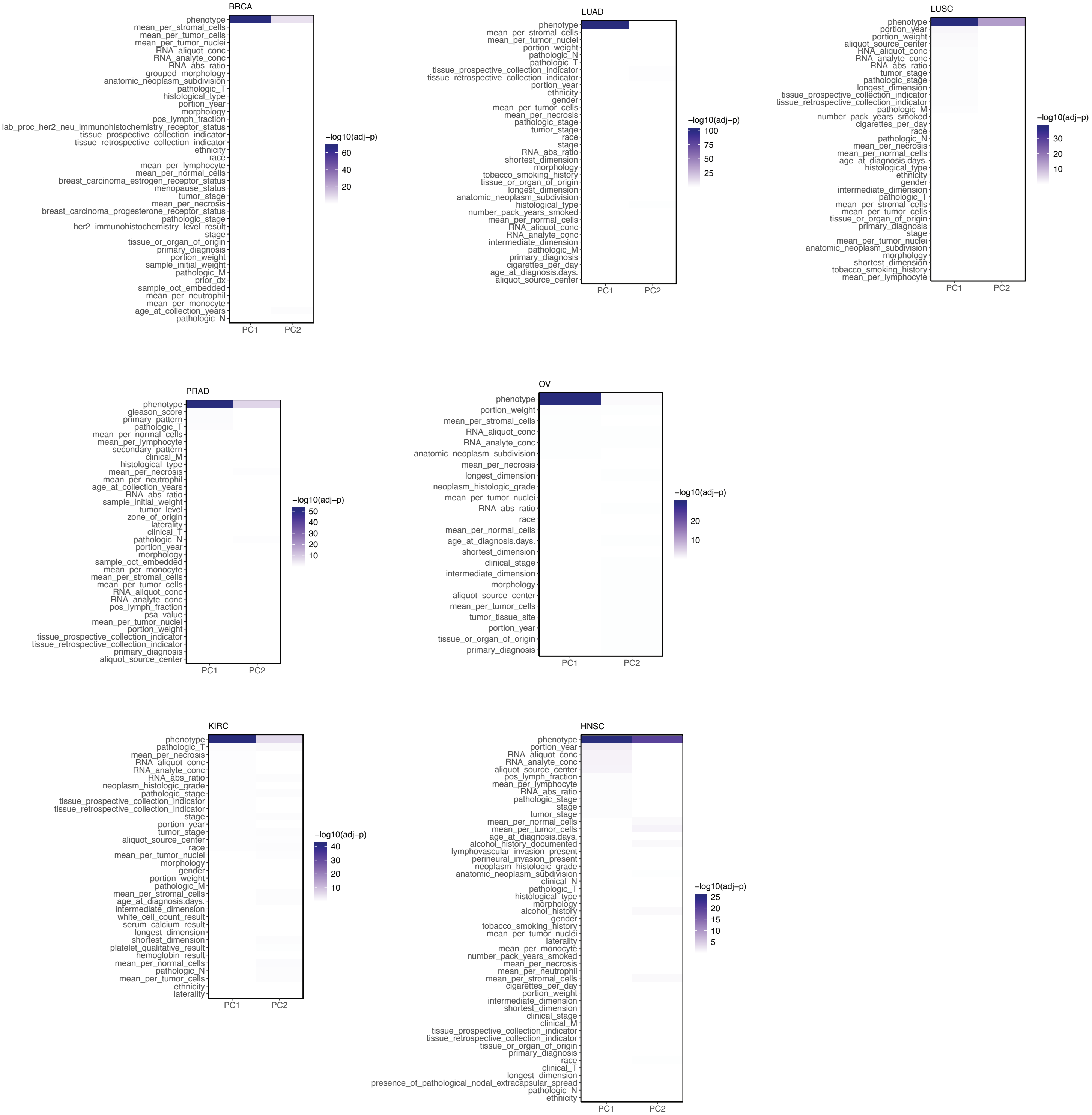


**Fig. S1. Differential editing not confounded by metadata.** Heatmaps of significance (log10-transformed adjusted p-values) of correlations between the top two principal components and E/M phenotype among metadata fields in each cancer type. Darker color indicates smaller p-value and stronger association.

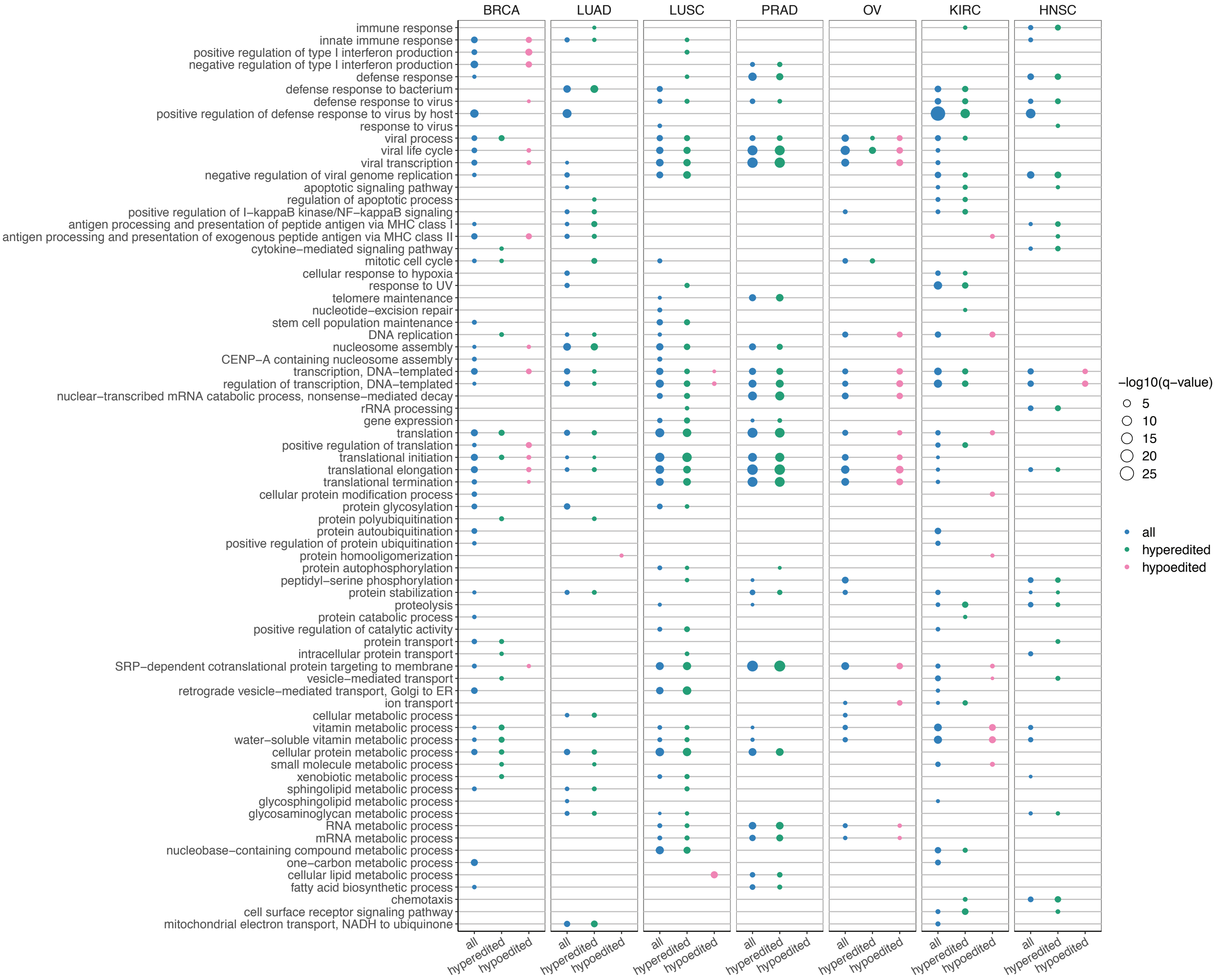


**Fig. S2. Gene ontology enrichment among differentially edited genes.** Significance of enrichment of gene ontology (GO) terms among all differentially edited genes (blue), only hyperedited genes (green) or only hypoedited genes (pink) of each cancer type. Point size represents the statistical significance of enrichment (log10-transformed adjusted p-value). Terms significantly enriched in at least two cancer types are shown. For cancer types with a global hyperediting trend in M tumors, GO enrichment among hyperedited genes is similar to that among all differentially edited genes. Likewise, for cancer types with a hypoediting trend (BRCA and OV), enrichment among hypoedited genes is similar to that among all differentially edited genes.

A

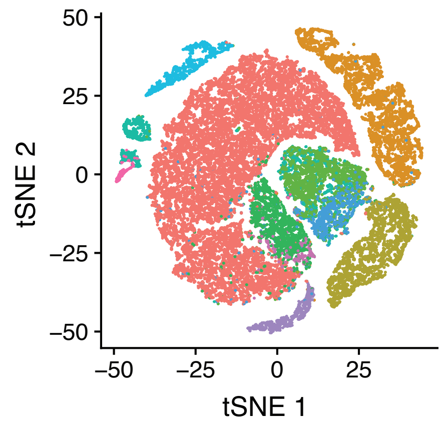


| Cluster | Matching cell types | Matching markers |
|---|---|---|
| ● (red) | T cell | CD3D |
| ● (orange) | Myeloid | LYZ,MARCO,CD68,FCGR3A |
| ● (olive) | Cancer | EPCAM |
| ● (light green) | Cancer | EPCAM |
| ● (green) | B cell | CD79A |
| ● (teal) | Cancer | EPCAM |
| ● (cyan) | Fibroblast | COL1A1,DCN,COL1A2,C1R |
| ● (blue) | B cell | CD79A |
| ● (purple) | Endothelial Cell | CLDN5,FLT1,CDH5,RAMP2 |
| ● (magenta) | Mast | TPSAB1,CPA3,HPGDS,KIT |
| ● (pink) | Alveolar; Epi; Cancer | FOLR1;CAPS,TMEM190,PIFO,SNTN;EPCAM |

B

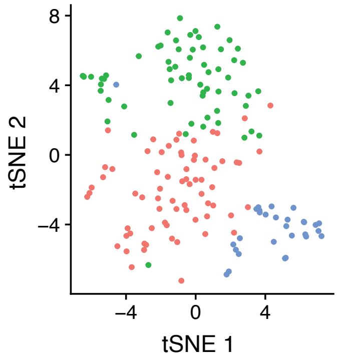


| Cluster | Matching cell types | Matching markers |
|---|---|---|
| ● (red) | NA | NA |
| ● (green) | Epithelial | CAPS,TMEM190,SNTN |
| ● (blue) | Alveolar | SFTPB |

C

| Cell Type | Count |
|---|---|
| T cell | 16354 |
| Cancer | 6526 |
| Myeloid | 4063 |
| B cell | 2770 |
| Fibroblast | 998 |
| Endothelial Cell | 771 |
| Mast | 285 |
| Epithelial | 62 |
| Alveolar | 29 |

D

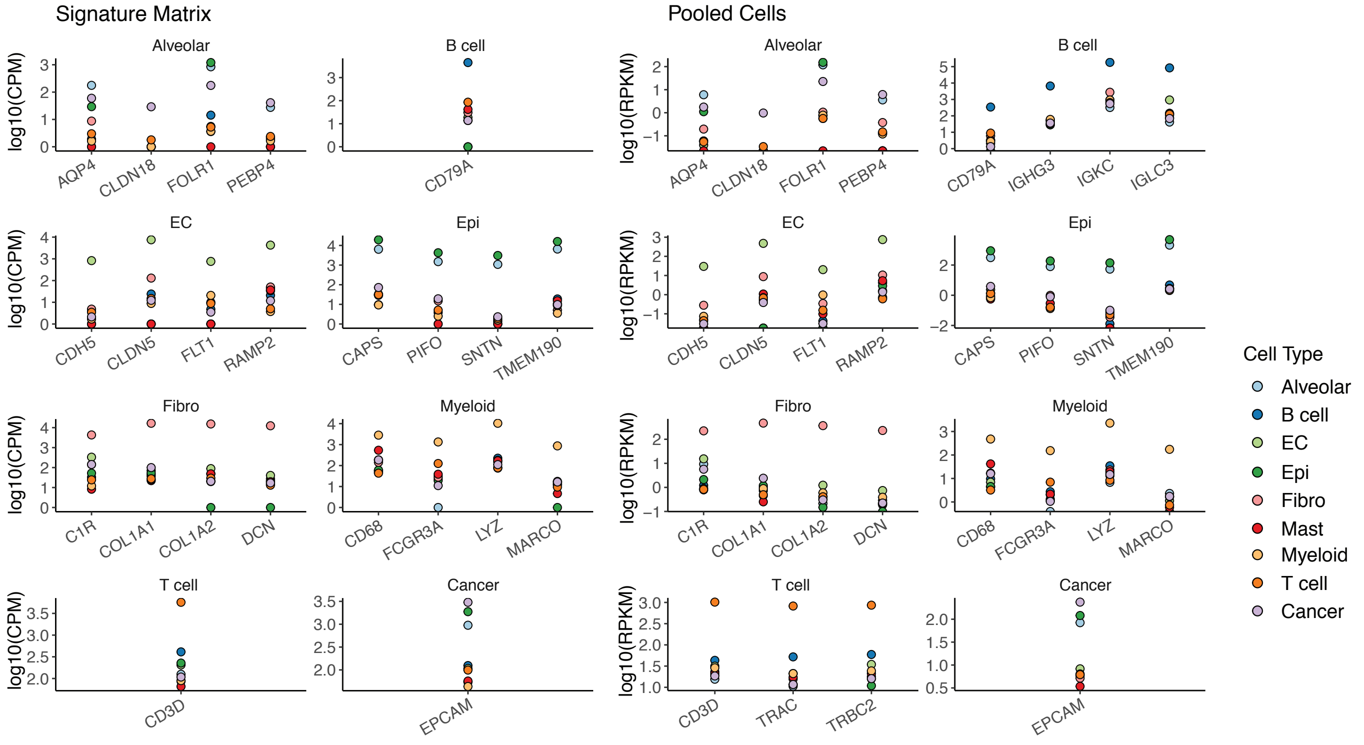


**Fig. S3. Clustering of single cells from three lung cancer tumors. A.** TSNE projection of cells based on expression profiles, with color indicating cluster identity (left). Cell types were assigned to clusters by matching differentially expressed genes of clusters to known cell type markers (right). **B.** TSNE projection of only cells from cluster 10 to further refine cell type assignment (left). Similar to **A**, cell types were labeled using differentially expressed genes that matched cell type markers (right). **C.** Counts of cells for each cell type after 2 rounds of clustering and cell type assignment (**A** and **B**). **D.** Log2-transformed expression values of marker genes across cell types. Signature matrix on the left indicates expression values assigned for each cell type by CIBERSORTx. On the right, Pooled Cells indicate that expression values were calculated from pooling reads from cells of the same type together.

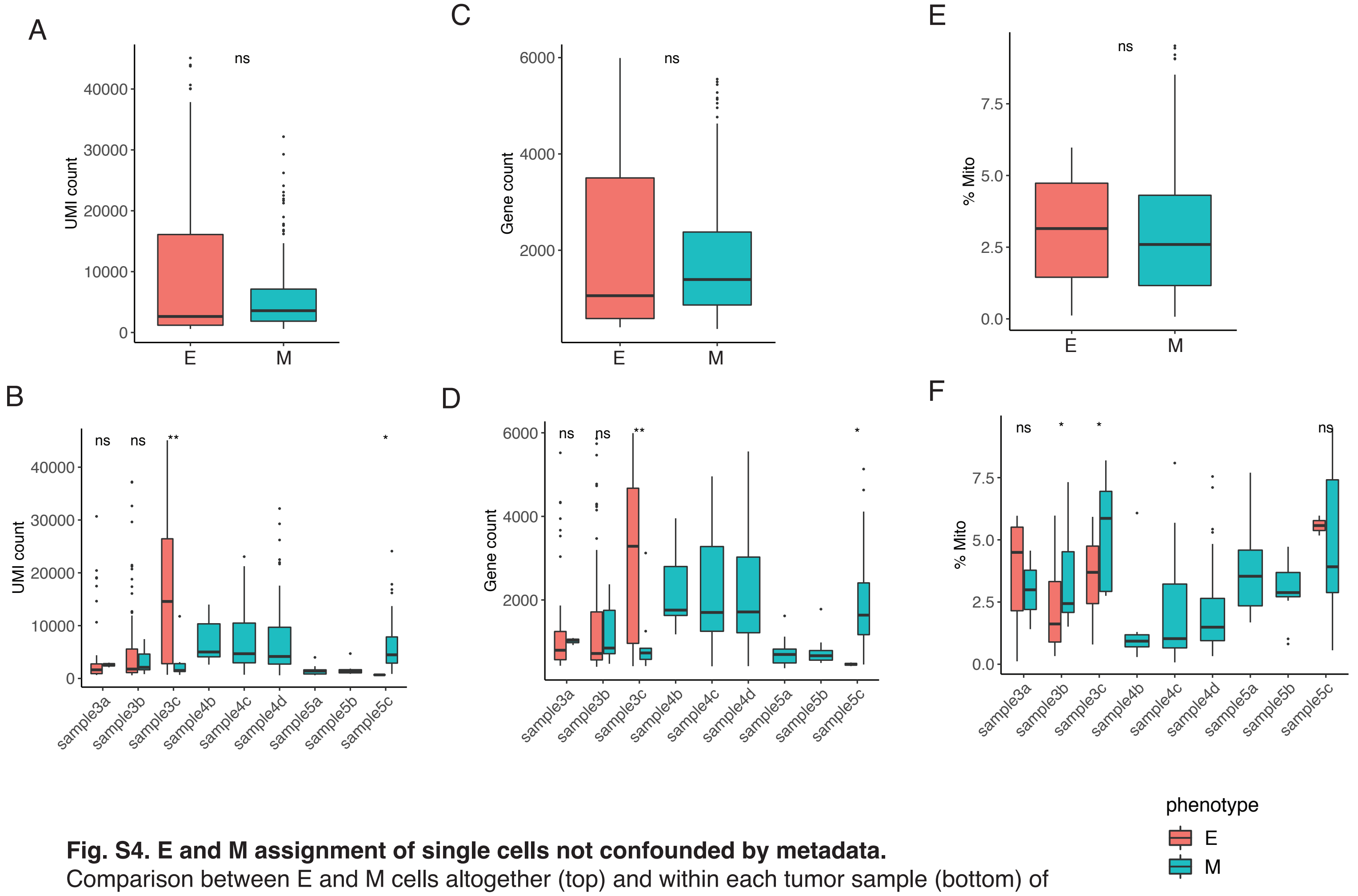


**Fig. S4. E and M assignment of single cells not confounded by metadata.**
Comparison between E and M cells altogether (top) and within each tumor sample (bottom) of metadata fields: UMI count (**A**-**B**), gene count (**C**-**D**), and percent of reads mapping to the mitochondrial genome (**E**-**F**). Metadata values were compared by Mann Whitney U tests, and significance of p-values are shown. ns: $p > 0.05$, * $p \leq 0.05$, ** $p \leq 0.01$.

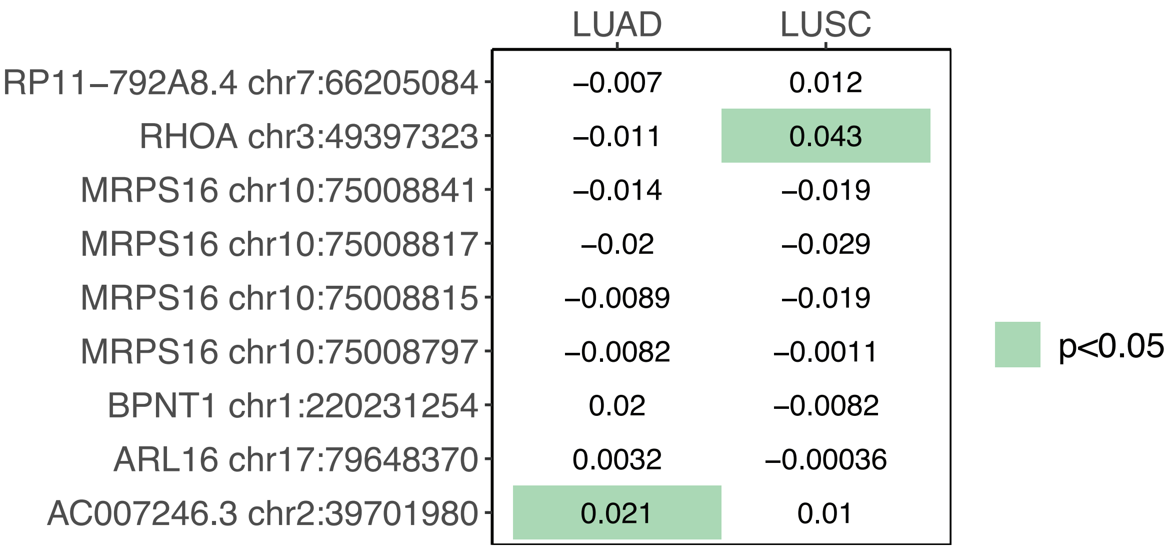


**Fig. S5. LUAD and LUSC tumor editing differences of differential sites identified from single cell RNA-seq analysis.** For each editing site, the difference in mean editing levels between M and E tumors (M - E) in each cancer type is listed. Green highlight indicates Wilcoxon p-value < 0.05.

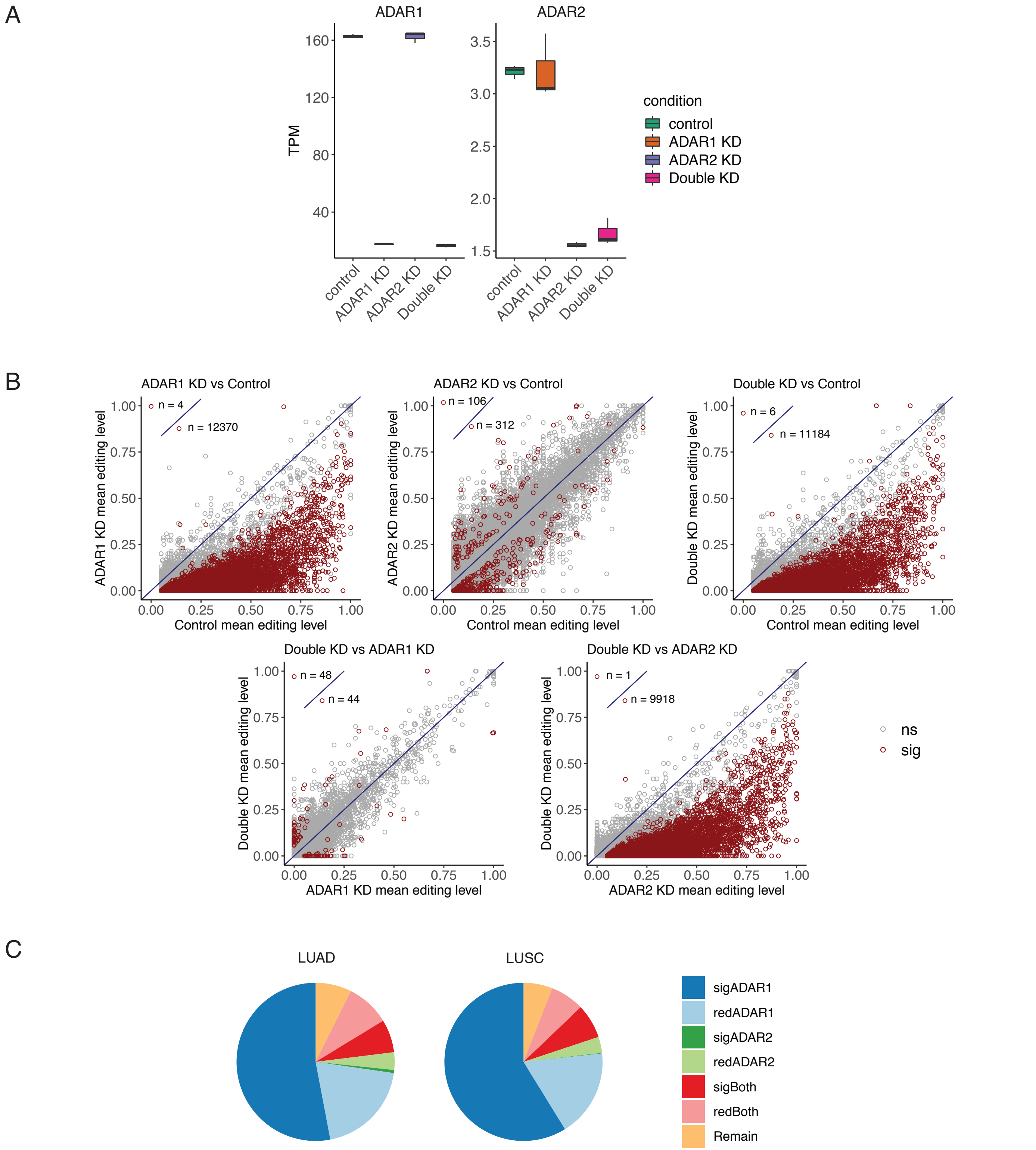


**Fig. S6. Altered editing upon knockdown of ADAR1, ADAR2, or both. A.** Distributions of mRNA expression of ADAR1 and ADAR2 under ADAR KD and control conditions. Expression levels were quantified as transcripts per million (TPM). **B.** Mean editing levels of testable sites in five comparisons between ADAR KD conditions or control experiment. Sites with significant editing differences between conditions are colored red, while gray represents nondifferential sites. Y=x line shown in blue. **C.** Proportions of lung cancer E-M differential sites that were also differential in ADAR KD conditions (compared to controls). sigADAR1: sites that were differential only in ADAR1 KD. sigADAR2: sites that were differential only in ADAR2 KD. sigBoth: sites that were differential in both ADAR1 KD and ADAR2 KD, or in double KD. The prefix ‘red’ indicates reduced editing level by at least 0.05 upon KD from control, but did not pass the statistical significance requirement. ‘Remain’: editing sites that were not significantly different or reduced across any comparison.

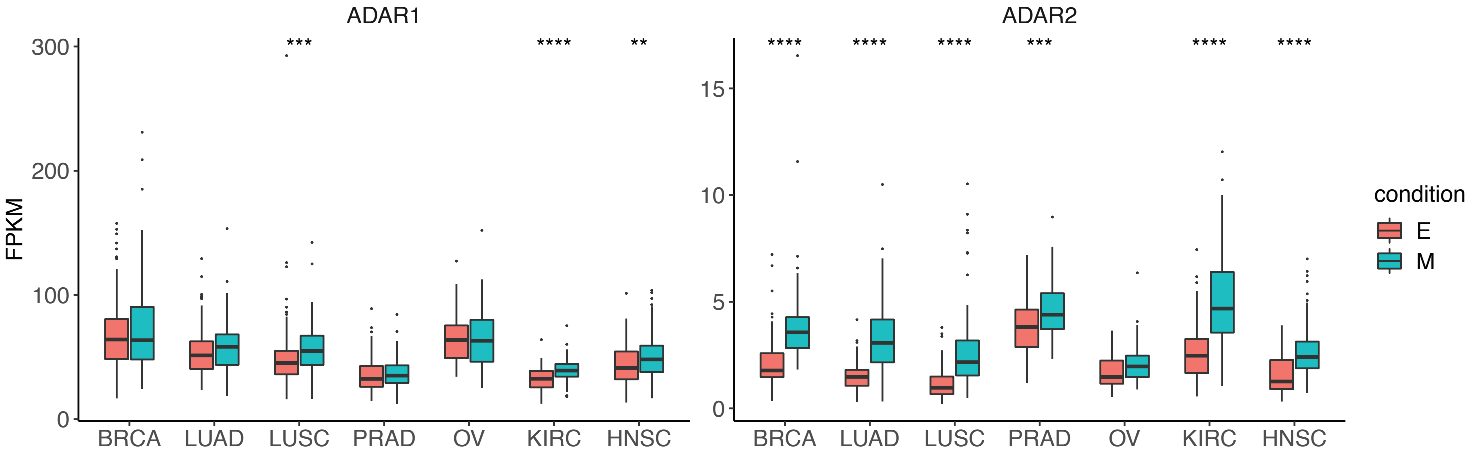


**Fig. S7. Expression of ADARs in E and M tumors.** Distributions of mRNA expression of ADAR1 (left) and ADAR2 (right) in E and M tumors across cancer types. Expression values, measured as Fragments Per Kilobase per Million mapped reads (FPKM), were compared by Mann Whitney U tests, and significance of p-values are shown. ** $p \leq 0.01$; *** $p \leq 0.001$; **** $p \leq 0.0001$.

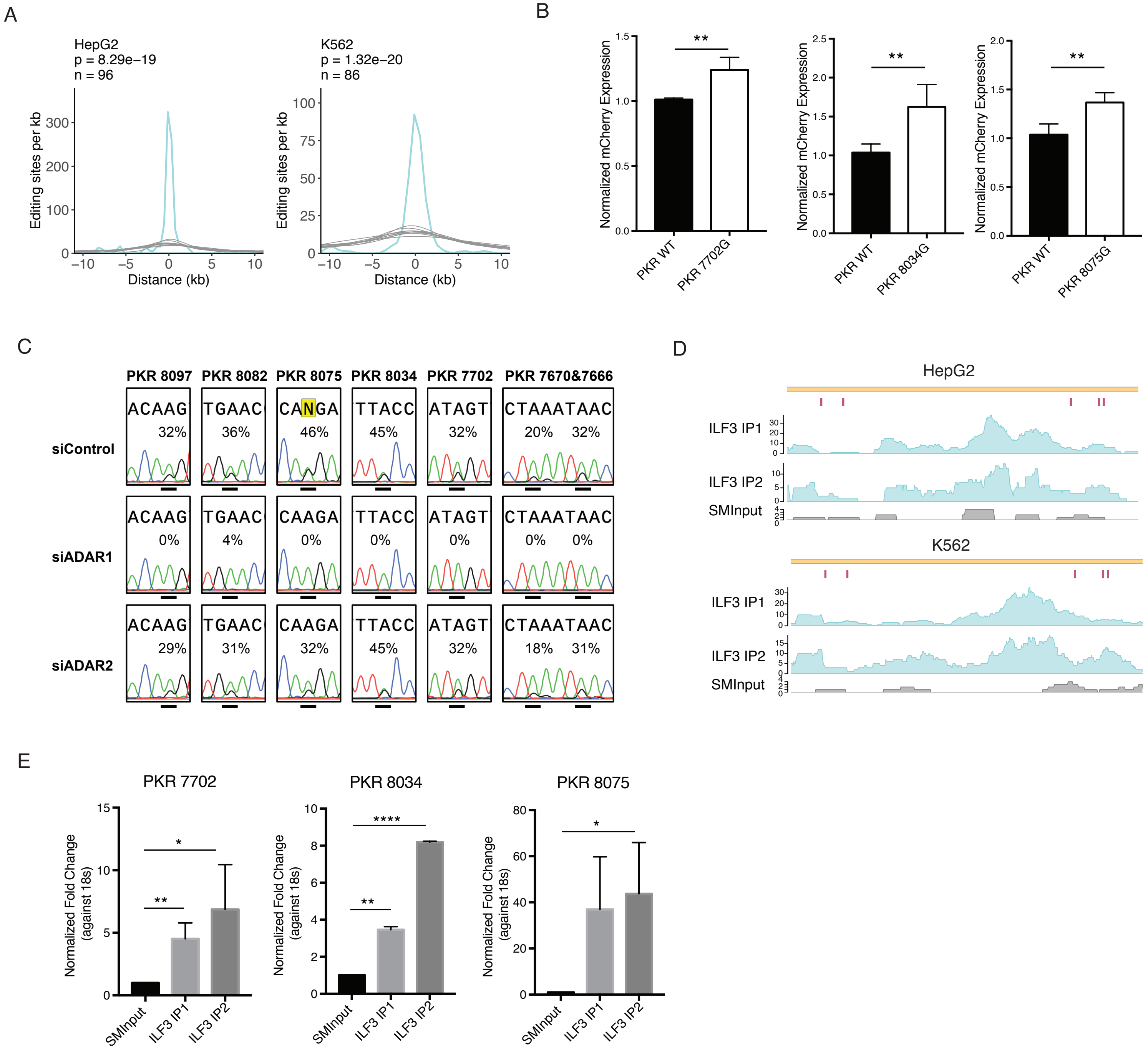


**Fig. S8. ILF3 binds closely to the differential editing sites in editing-expression correlated genes.**
**A.** Histogram of distances between differential editing sites in editing-correlated genes and the closest ILF3 eCLIP peaks in HepG2 and K562 cells (turquoise), up to 10 kb. Gray curves represent distances for 10 sets of randomly picked A's in the same genes as differential editing sites. Number of differential editing sites is given by n for each cell line. P-value was calculated by comparing the area under the curve (AUC) of the distance distribution for differential editing sites to a normal distribution fit to the AUC values of 10,000 sets of random gene-matched A's. **B.** Normalized mCherry expression for nonedited or edited versions of sites in the 3'UTR of PKR in A549 cells. Five biological replicates were performed. Normalized expression values were compared between edited and nonedited versions by two-sided t-test. **p<0.01.
**C.** Editing levels of PKR 3'UTR editing sites in siControl, siADAR1 and siADAR2 A549 cells measured by Sanger sequencing. The peak signals of A and G nucleotides were measured by 4Peaks for editing level calculation (G/(A+G)). The editing level of each editing site (underlined) is shown in the graph. **D.** Read coverage of ILF3 eCLIP-seq in HepG2 and K562 cells for two biological replicates (ILF3 IP1 and ILF3 IP2, turquoise) and size-matched input (SMInput, gray) in each cell line. The five validated 3' UTR editing sites affecting PKR mRNA abundance in A549 cells are labeled in magenta.
**E.** Validation of PKR eCLIP signal overlapping three editing sites. PKR expression was measured by qRT-PCR in the IP or SMInput samples and normalized against the expression of 18s rRNA. Three technical replicates were performed (other than two replicates for 8034). P-value calculated by t-test. *p<0.05, **p<0.01, ****p<0.0001.

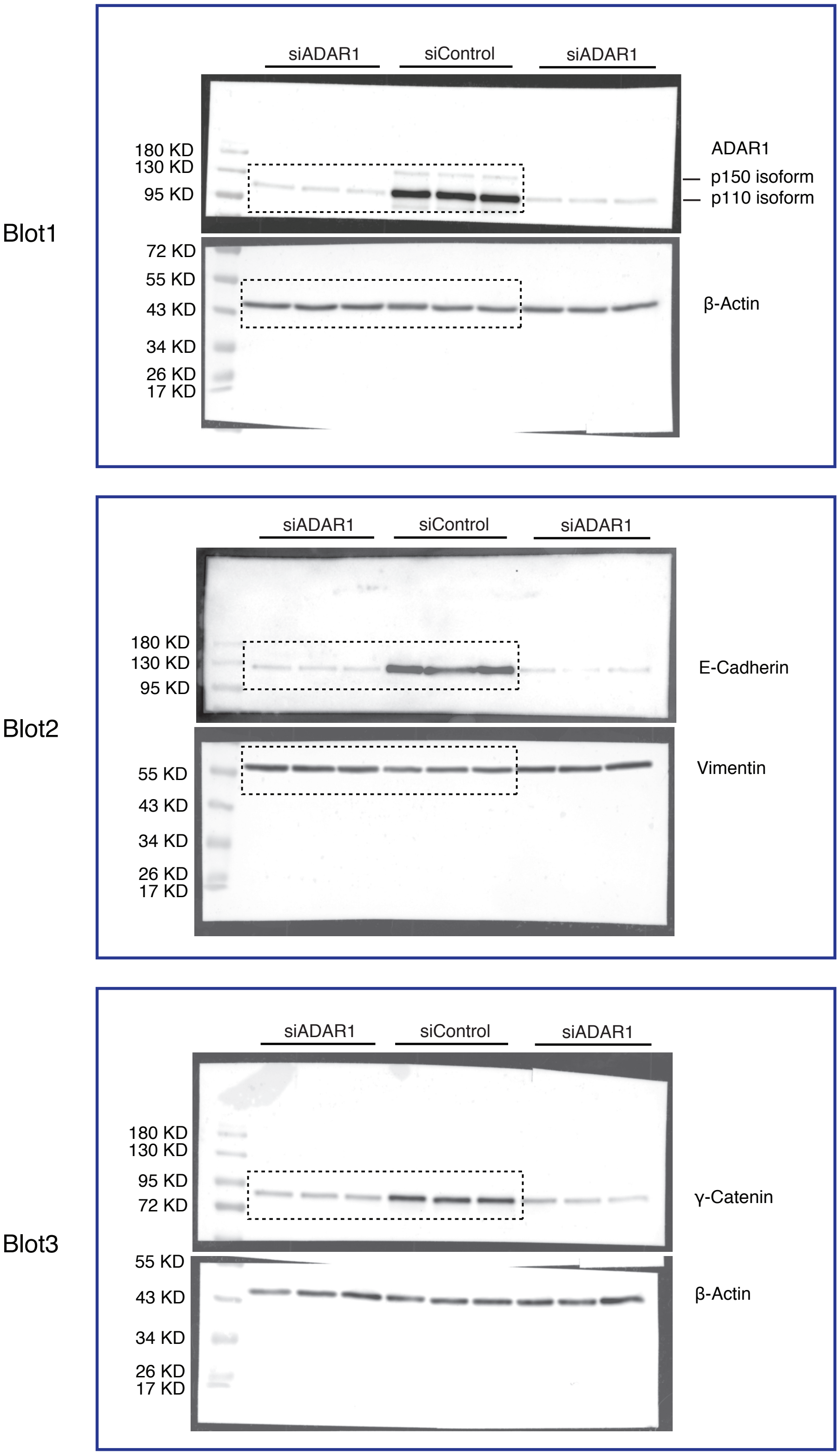


**Fig. S9. Uncropped western blot images for Figure 4A.** The original blots were cut to incubate with different antibodies and imaged separately. The images from the same blot were grouped by the large blue rectangles.

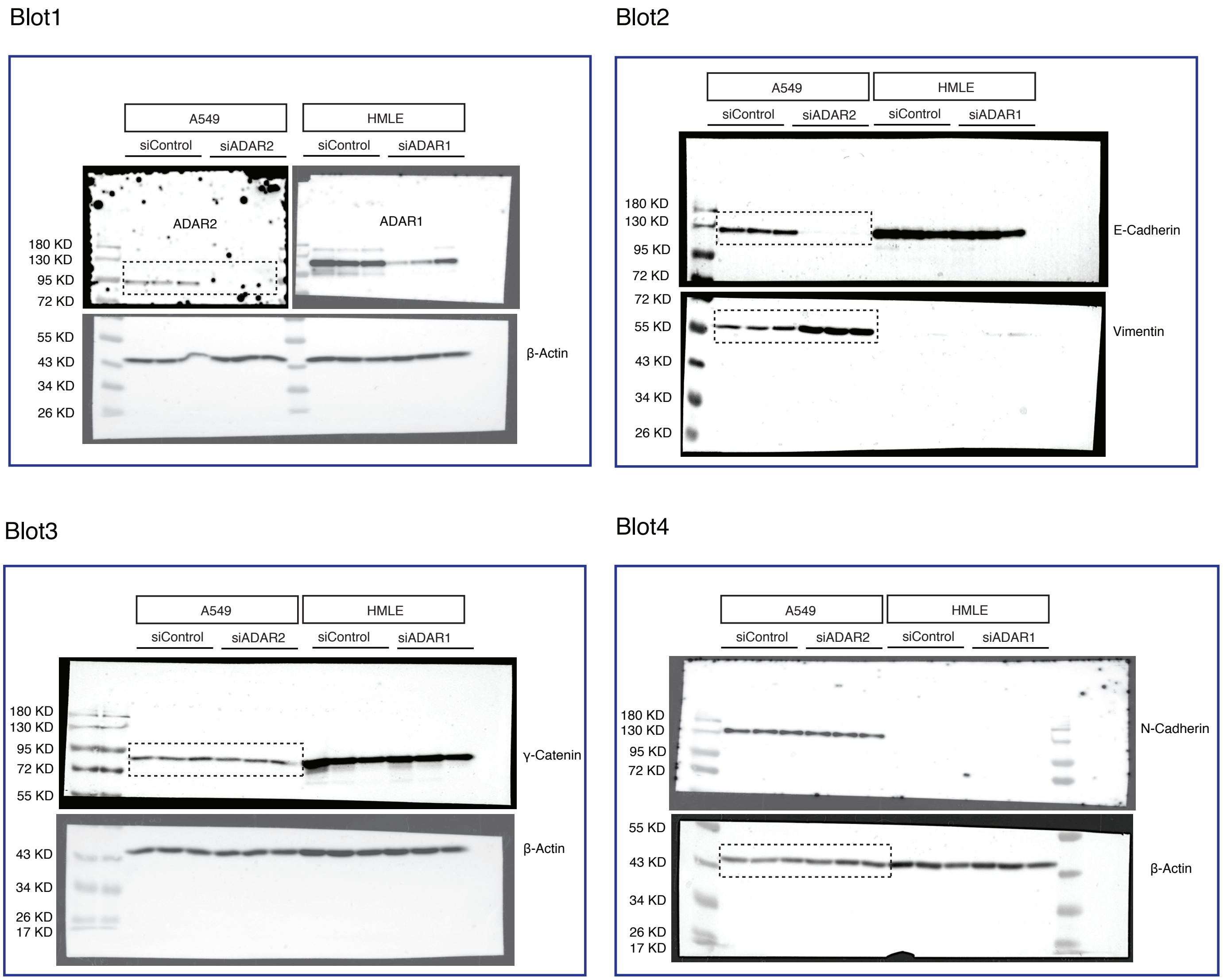


**Fig. S10. Uncropped western blot images for Figure 4B.** The original blots were cut to incubate with different antibodies and imaged separately. The images from the same blot were grouped by the large blue rectangles.

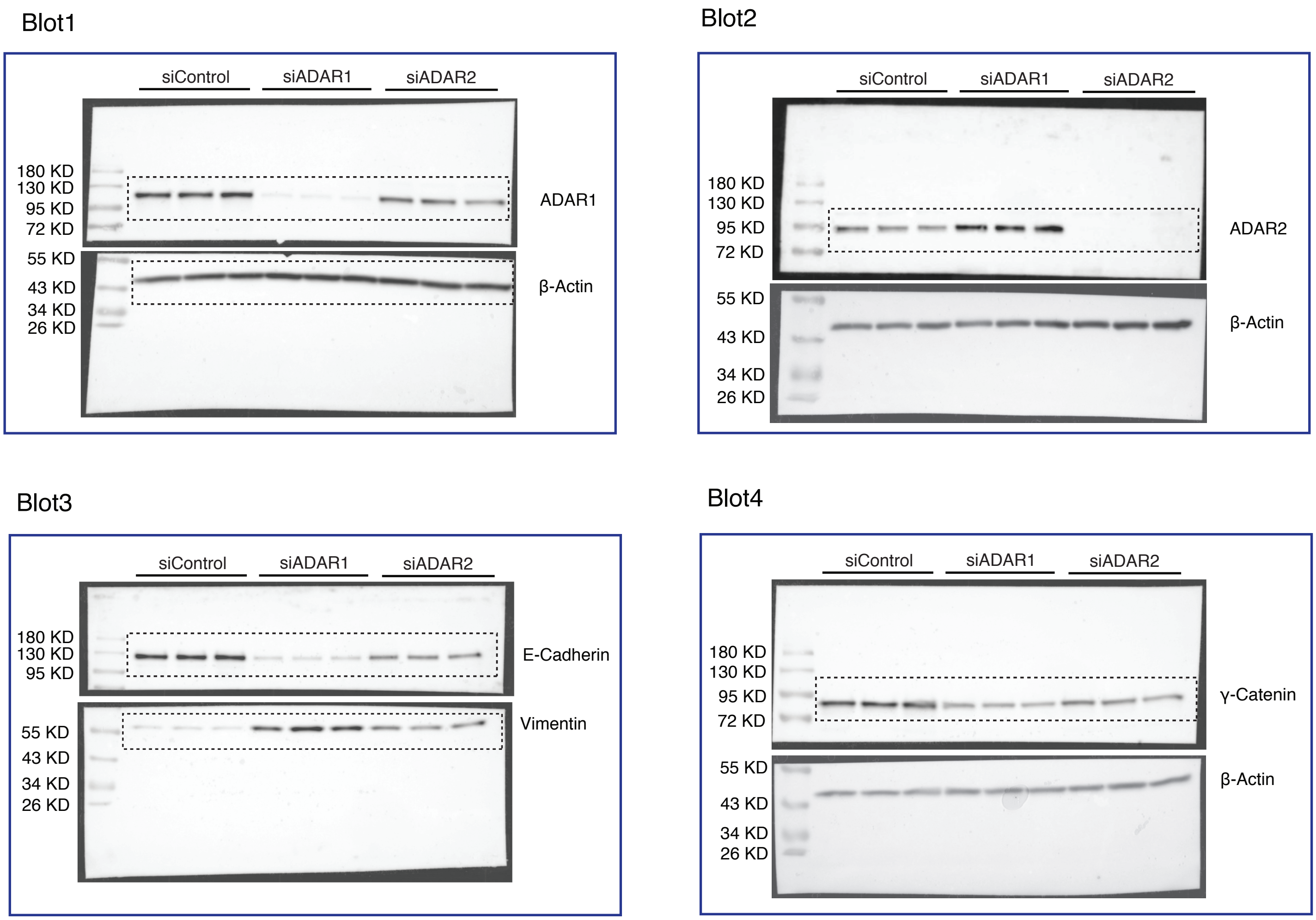


**Fig. S11. Uncropped western blot images for Figure 4C.** The original blots were cut to incubate with different antibodies and imaged separately. The images from the same blot were grouped by the large blue rectangles.

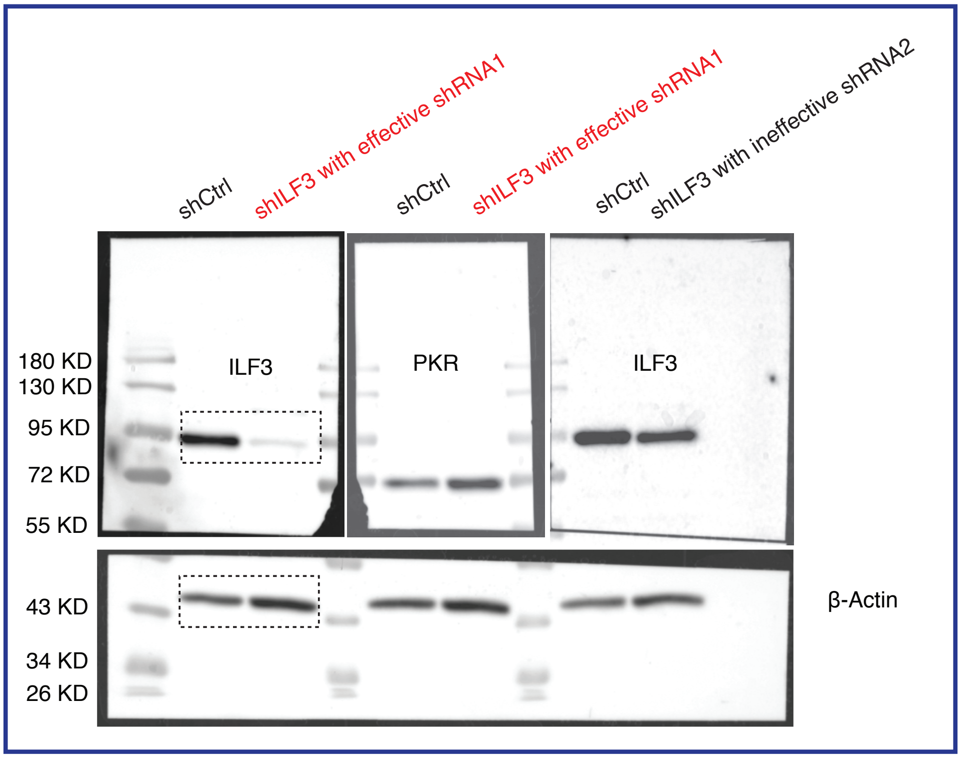


**Fig. S12. Uncropped western blot images for Figure 7A.** Two different shRNAs were used to generated the ILF3 KD A549 cells. The frist shRNA(highlighted in red) was more effective than the second shRNA and was used for the reporter assays in this study. The original blots were cut to incubate with different antibodies and imaged separately. The images from the same blot were grouped by the large blue rectangle.

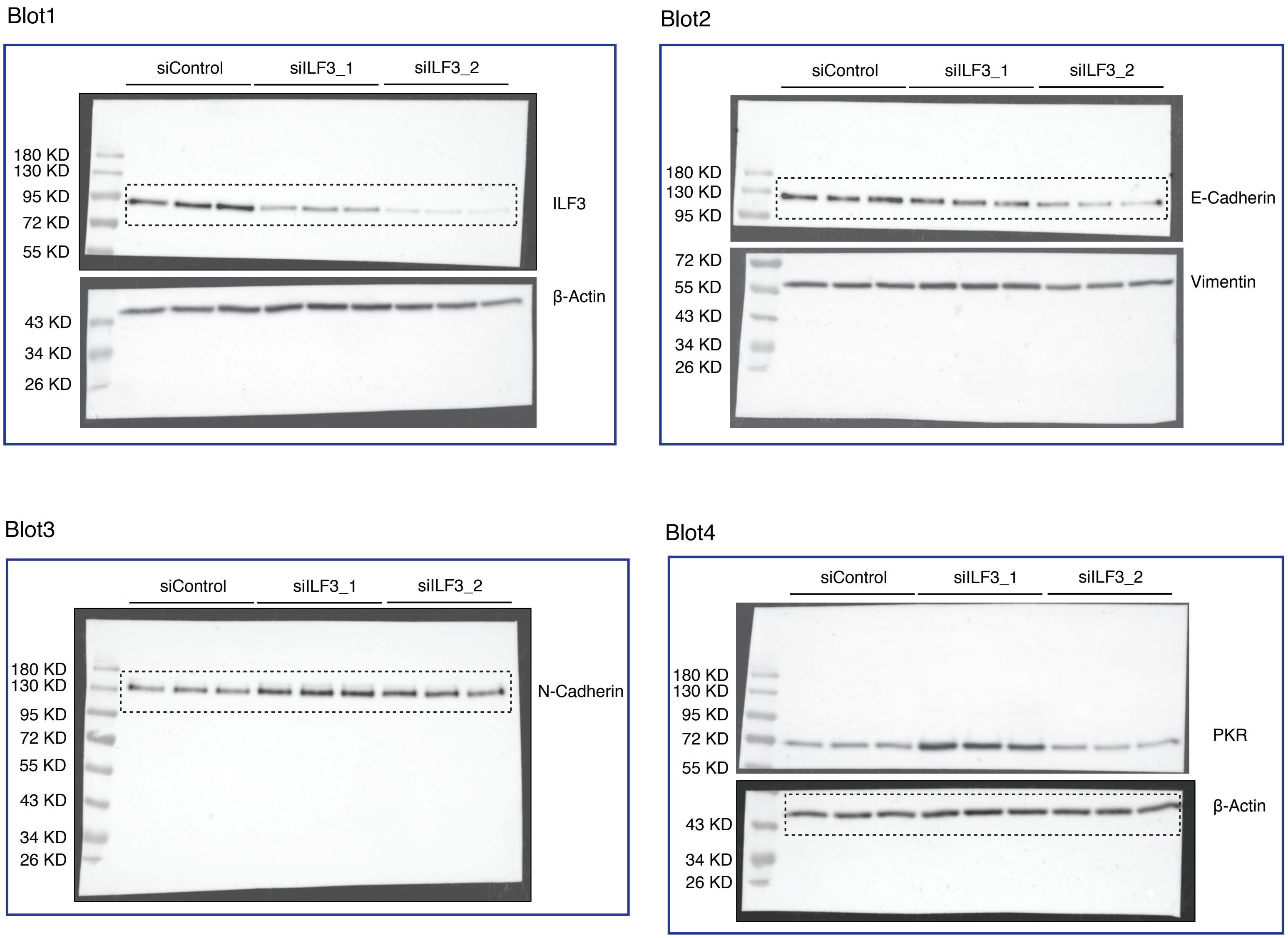


**Fig. S13. Uncropped western blot images for Figure 7D.** The original blots were cut to incubate with different antibodies and imaged separately. The images from the same blot were grouped by the large blue rectangles.
